# Effects of Stool Sample Preservation Methods on Gut Microbiota Biodiversity: New Original Data and Systematic Review with Meta-Analysis

**DOI:** 10.1128/spectrum.04297-22

**Published:** 2023-04-24

**Authors:** Xin-meng Li, Xiao Shi, Yao Yao, Yi-cun Shen, Xiang-ling Wu, Ting Cai, Lun-xi Liang, Fen Wang

**Affiliations:** a Department of Gastroenterology, the Third Xiangya Hospital, Central South University, Changsha, Hunan, China; b Hunan Key Laboratory of Non-resolving Inflammation and Cancer, Central South University, Changsha, Hunan, China; c Department of Dermatology, Anhui Provincial Hospital, The First Affiliated Hospital of USTC, Division of Life Science and Medicine, University of Science and Technology of China, Hefei, China; d Department of Gastroenterology, Zhangjiajie People’s Hospital, Zhangjiajie, Hunan, China; e Department of Gastroenterology, The Affiliated Changsha Central Hospital, Hengyang Medical School, University of South China, Changsha, China; Taichung Veterans General Hospital

**Keywords:** stool, biological sample, gut microbiome, preservation

## Abstract

Here, we aimed to compare the effects of different preservation methods on outcomes of fecal microbiota. We evaluated the effects of different preservation methods using stool sample preservation experiments for up to 1 year. The stool samples from feces of healthy volunteers were grouped based on whether absolute ethanol was added and whether they were hypothermically preserved. Besides, we performed a systematic review to combine current fecal microbiota preservation evidence. We found that *Proteobacteria* changed significantly and *Veillonellaceae* decreased significantly in the 12th month in the room temperature + absolute ethanol group. The four cryopreservation groups have more similarities with fresh sample in the 12 months; however, different cryopreservation methods have different effects on several phyla, families, and genera. A systematic review showed that the Shannon diversity and Simpson index of samples stored in RNAlater for 1 month were not statistically significant compared with those stored immediately at −80°C (*P* = 0.220 and *P* = 0.123, respectively). The −80°C refrigerator and liquid nitrogen cryopreservation with 10% glycerine can both maintain stable microbiota of stool samples for long-term preservation. The addition of absolute ethanol to cryopreserved samples had no significant difference in the effect of preserving fecal microbial characteristics. Our study provides empirical insights into preservation details for future studies of the long-term preservation of fecal microbiota. Systematic review and meta-analysis found that the gut microbiota structure, composition, and diversity of samples preserved by storage methods, such as preservation solution, are relatively stable, which were suitable for short-term storage at room temperature.

**IMPORTANCE** The study of gut bacteria has become increasingly popular, and fecal sample preservation methods and times need to be standardized. Here, we detail a 12-month study of fecal sample preservation, and our study provides an empirical reference about experimental details for long-term high-quality storage of fecal samples in the field of gut microbiology research. The results showed that the combination of −80°C/liquid nitrogen deep cryopreservation and 10% glycerol was the most effective method for the preservation of stool samples, which is suitable for long-term storage for at least 12 months. The addition of anhydrous ethanol to the deep cryopreserved samples did not make a significant difference in the preservation of fecal microbiological characteristics. Combined with the results of systematic reviews and meta-analyses, we believe that, when researchers preserve fecal specimens, it is essential to select the proper preservation method and time period in accordance with the goal of the study.

## INTRODUCTION

The gut microbiota has become an important health-monitoring and regulation topic for people in recent years (1). The gut microbiota consists of trillions of bacteria, and alterations in the gut microbiota have been linked to disease states, such as infection, inflammatory bowel disease, obesity, and diabetes. In addition, there is mounting evidence suggesting that the composition of gut microbiota is related to gut functions (e.g., bloating, cramping, and constipation) and some subhealthy life states (anxiety and stress) (2). The most accessible resource for studying human and animal microbiota is feces. High-throughput sequencing analysis of DNA extracted from feces to study the gut microbiota has been shown to be an alternative to human colon microbiota (3). Since the previously used bacterial culture methods failed to identify 60 to 70% of the common gut bacteria, microbiome research has progressed relatively quickly in the last decade, driven by the development and implementation of novel sequencing technologies and bioinformatics techniques. Meanwhile, the dramatic reduction in sequencing costs has facilitated the integration of microbiome studies into large-scale epidemiological studies. The characterization of microbial communities helps to elucidate the rich and diverse microbial landscapes in humans and animals as well as the great variation among individuals, and in addition to the identification of the microbiota present, the function of the microbiota can be expressed by metagenomic sequencing (4, 5).

However, in scientific research, especially clinical experiments, it is difficult to obtain a large number of samples simultaneously, so it is required to collect biological samples across time or even geography. The extension of freezing and storage time or the increase of freeze-thaw cycles may lead to the instability of sample biological information. Different preservation methods are selected according to the clinical needs of various experiments, and the influence of pre-experimental variables on experimental results and clinical efficacy is prevented as much as possible. The collection of biological samples has changed from a temporary collection of samples according to the needs of research projects to standardized, professional, and batch collection. In this study, we describe the mechanisms involved in the preservation of microorganisms and cells by different preservation methods, review the common preservation methods regarding stool samples, explore the main factors affecting their preservation effects, and provide an outlook on the feasible future ways of preserving stool samples. In addition, our study summarizes the impact of preservation methods on the results of intestinal microbiota in stool samples from healthy volunteers via employing systematic review and meta-analysis.

## RESULTS

### Original data.

**(i) 16S rRNA gene sequencing.** The sequencing yielded mean counts of 56,355 valid sequences for subsequent analysis. After chimera removal, the effective rate of all data is more than 99%. The number of sequences that could not be classified into operational taxonomic units (OTUs) was 26,728. The average value of OTUs was 329.

**FIG 1:**
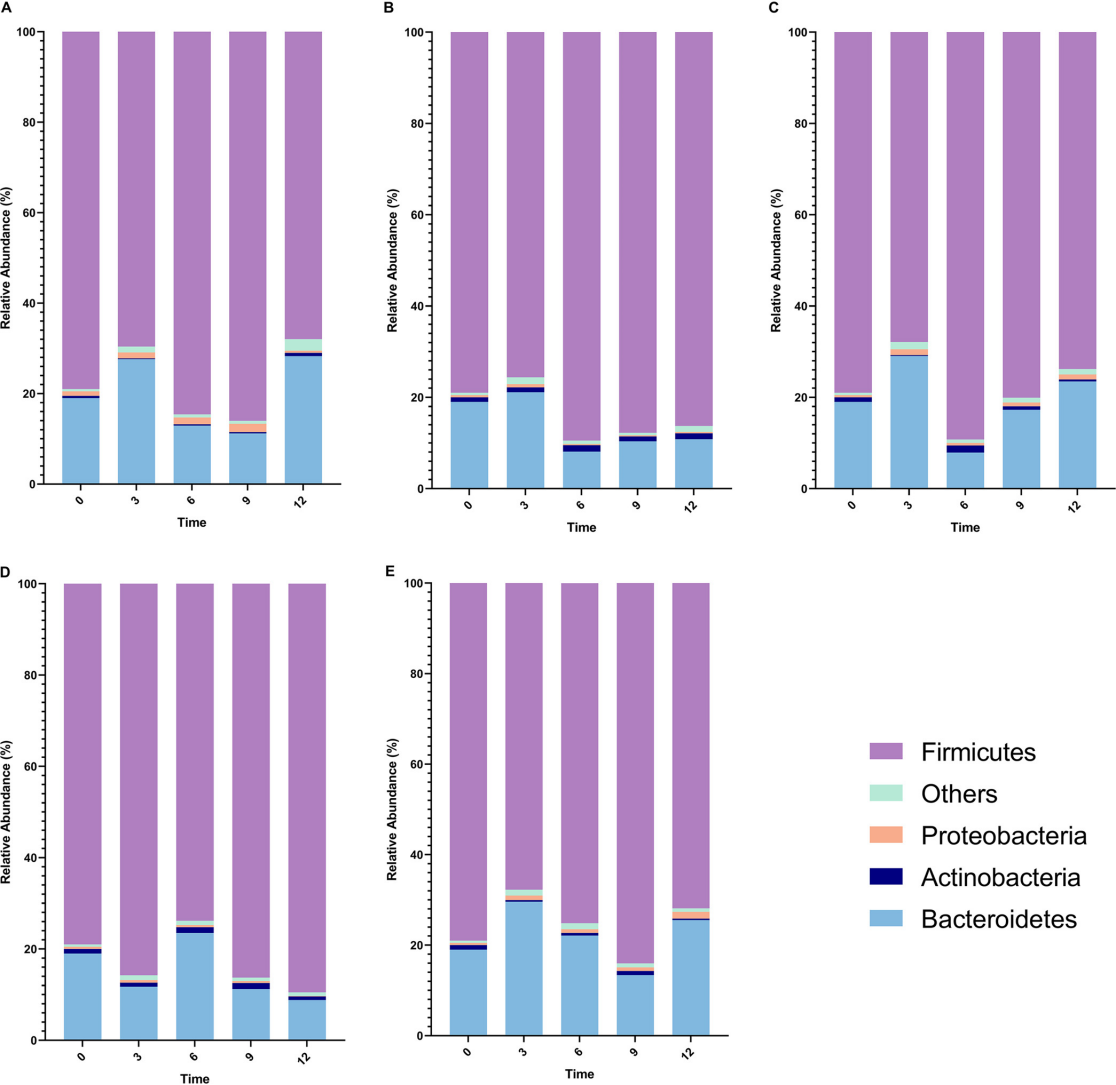
Relative abundance of bacterial phylum according to different storage conditions. (A) Room temperature + absolute ethanol group. (B) The −80°C refrigerator group. (C) The −80°C refrigerator + absolute ethanol group. (D) Liquid nitrogen tank group. (E) Liquid nitrogen + absolute ethanol group.

**(ii) Distribution of fecal microbiota.** At the phylum level, the top four most abundant bacteria were *Firmicutes*, *Bacteroidetes*, *Proteobacteria*, and *Actinobacteria*. In the 12th month, the abundance of *Proteobacteria* in the room temperature (RT) + absolute ethanol group significantly increased (*P < *0.05), while the bacterial abundance was most stable in the liquid nitrogen + absolute ethanol group (Fig. 1).

**FIG 2:**
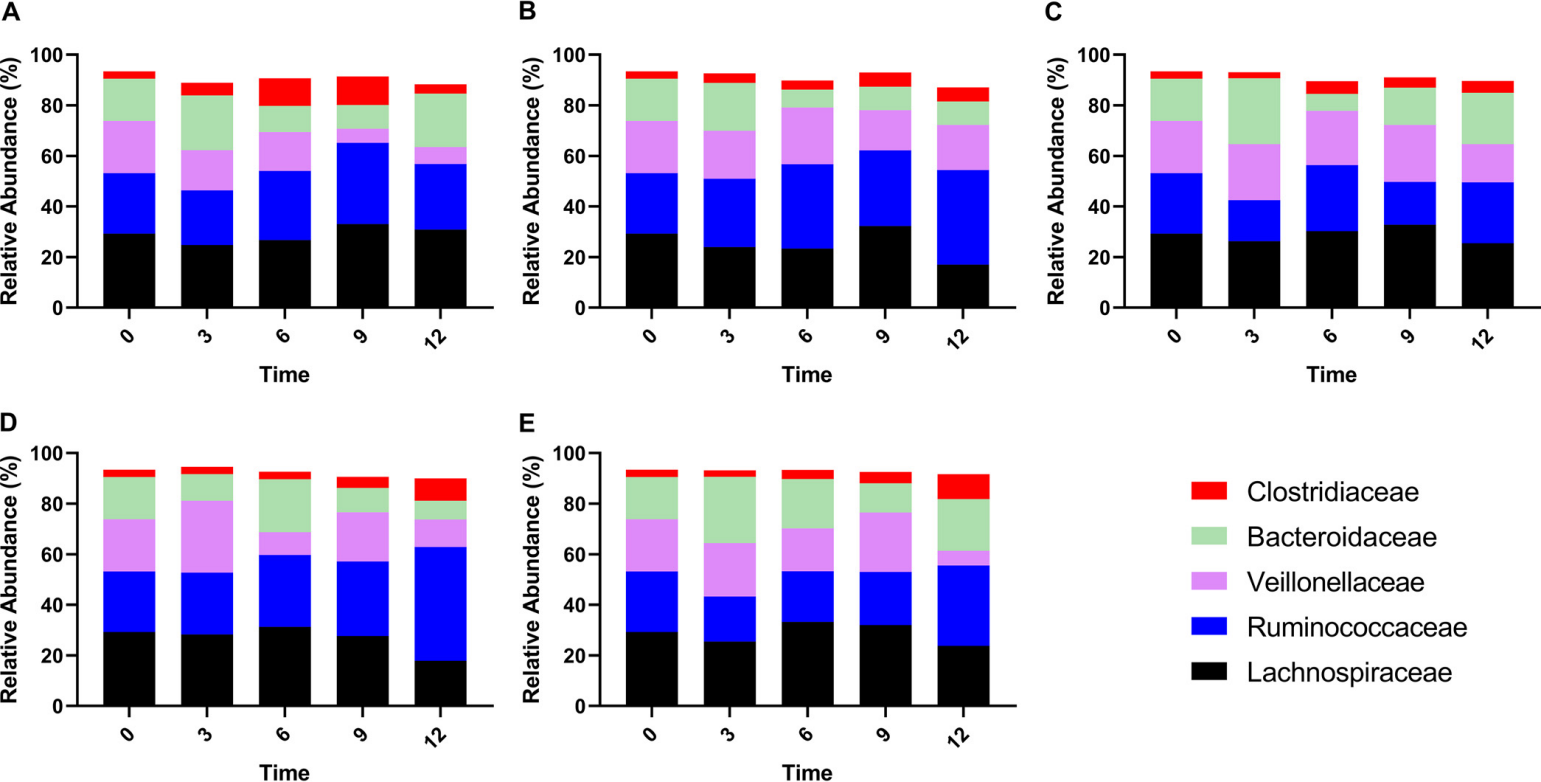
Relative abundance of bacterial families according to different storage conditions. (A) Room temperature + absolute ethanol group. (B) The −80°C refrigerator group. (C) The −80°C refrigerator + absolute ethanol group. (D) Liquid nitrogen tank group. (E) Liquid nitrogen + absolute ethanol group.

The top five most prevalent families detected in all samples were *Bacteroidaceae*, *Lachnospiraceae*, *Ruminococcaceae*, *Veillonellaceae*, and *Clostridiaceae*. In the room temperature + absolute ethanol group, the abundance of *Veillonellaceae* decreased compared with fresh samples (*P < *0.05 in the 12th month). The abundance of *Lachnospiraceae* was relatively stable in the five storage methods (*P ≥ *0.05), and the abundance of *Clostridiaceae* was relatively stable in the −80°C refrigerator group and −80°C refrigerator + absolute ethanol group (*P > *0.05) (Fig. 2).

**FIG 3:**
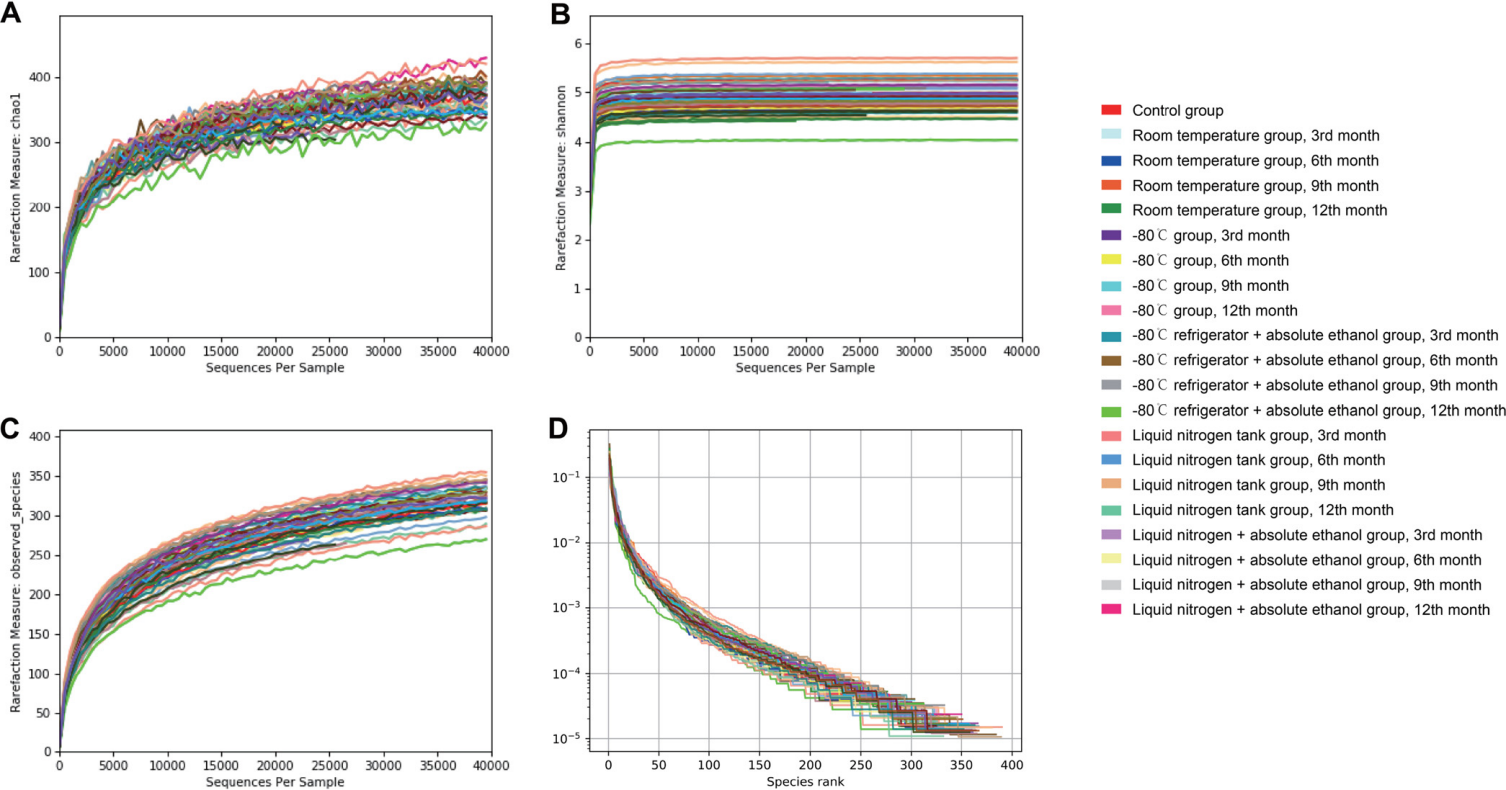
Diversity curves of sample microbial communities under different preservation methods. (A) Species richness index. (B) Shannon diversity index. (C) Simpson index. (D) Rank abundance curve.

**(iii) Alpha diversity analysis.** In the room temperature + absolute ethanol group (including four curves in Fig. 3), the microbial diversity and abundance decreased compared with those of other groups, and the decline is more evident with prolonged storage time (Fig. 3).

**FIG 4:**
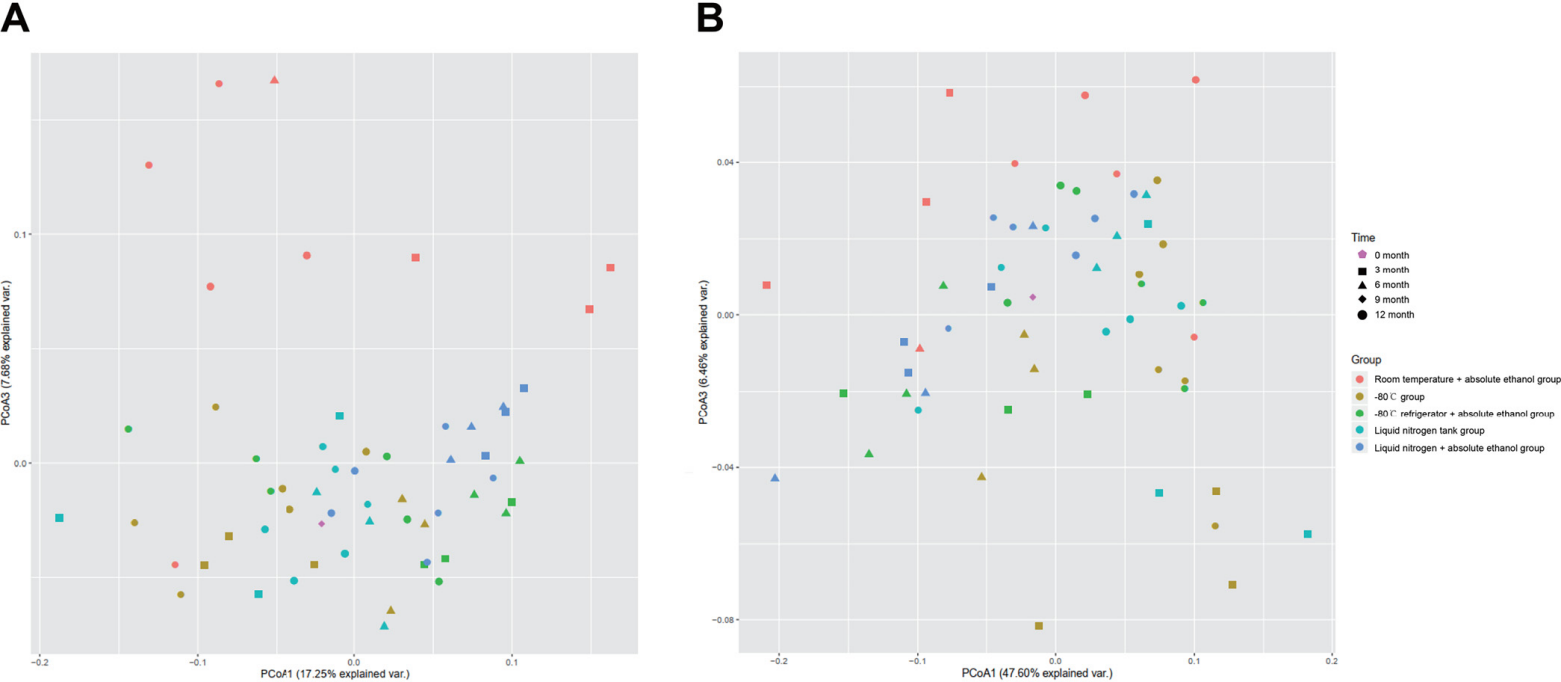
PCoA plot based on unweighted and weighted UniFrac distance matrices of samples in in different storage methods.

**(iv) Beta diversity analysis.** The differences between samples can be observed by the distance of sample points in Fig. 4. The closer the sample points are to each other indicates that the microbial community composition is more similar between samples. As shown in Fig. 4A (unweighted UniFrac distance) and Fig. 4B (weighted UniFrac distance), all samples in the room temperature + absolute ethanol group were in different quadrants and deviated from the fresh sample group, indicating that there were differences in microbial community structure between these two groups.

**(v) Differential abundance analysis between groups.** We found a statistically significant difference in the increase of *Eubacterium* compared with fresh samples at month 3 in the room temperature group (*P < *0.01), and a statistically significant difference in the decrease of *Desulfovibrio* and *Dorea* and a decrease of *Prevotella* but no statistically significant difference (*P > *0.05). In the sixth month in the room temperature group, the decrease of *Eubacterium* (*P < *0.05), *Desulfovibrio* (*P < *0.01), *Prevotella* (*P < *0.01), and *Megamonas* (*P < *0.05) compared with fresh samples was statistically significant. At the ninth month in the room temperature group, compared with fresh samples, *Eubacterium* (*P < *0.05), *Megamonas* (*P < *0.01), *Clostridium* (*P < *0.05), and *Adlercreutzia* (*P < *0.05) had a statistically significant reduction. At month 12 in the room temperature group, there was a statistically significant difference in the decrease of *Eubacterium* (*P < *0.05), *Desulfovibrio* (*P < *0.01), *Clostridium* (*P < *0.05), and *Adlercreutzia* (*P < *0.05) compared with fresh samples. For the −80°C freezer group, compared with fresh samples, *Rothia* and *Slackia* increased in 3 months, while *Prevotella* and *Fusobacterium* decreased in 3 months. Compared with the fresh samples, the abundance of *Catenibacterium* and *Slackia* increased, while *Actinomyces* and *Adlercreutzia* were reduced in the sixth month, but there was no statistical difference (*P > *0.05). Furthermore, there were no differences between the other groups and between the groups and fresh samples.

### Systematic review and meta-analysis.

**FIG 5:**
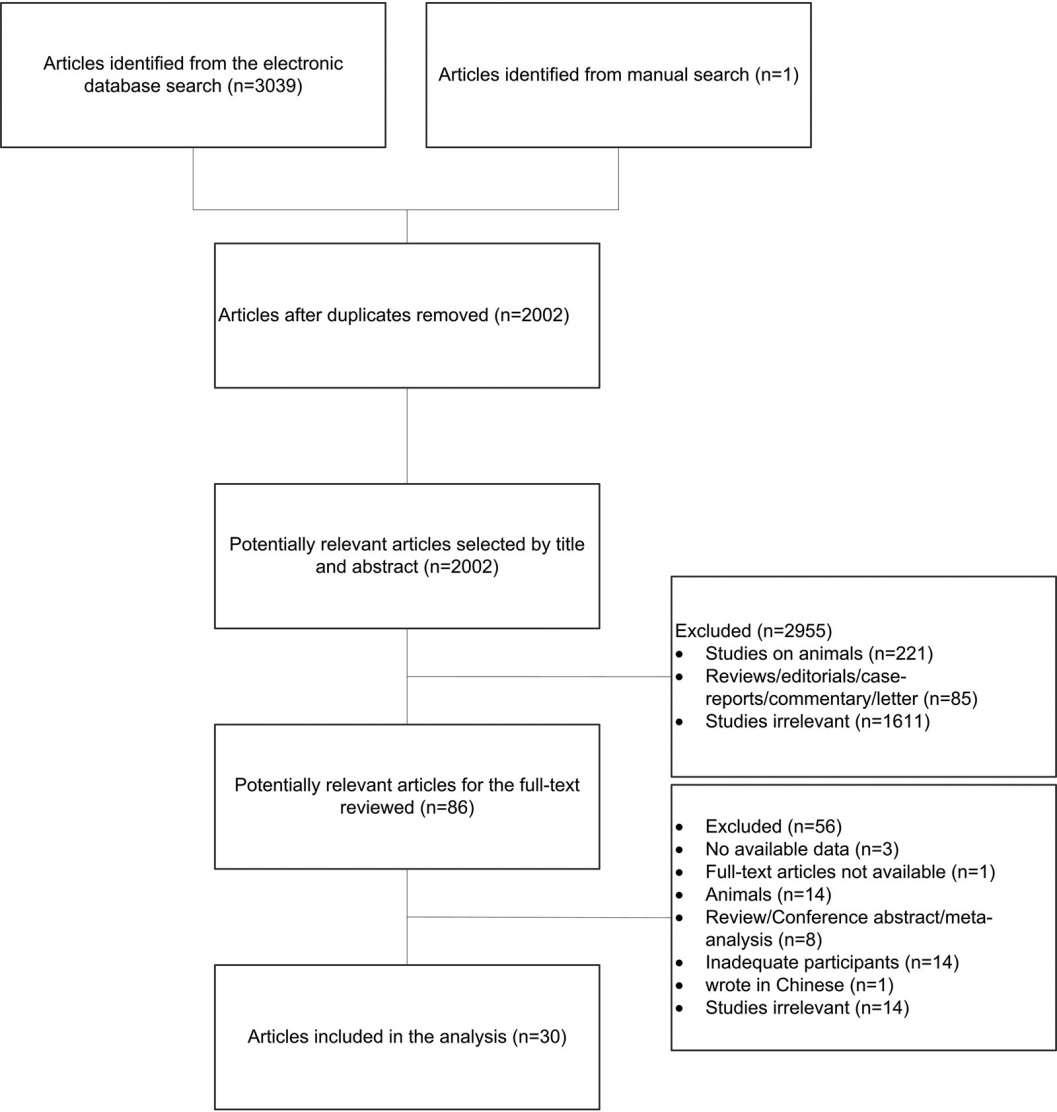
Flow chart for retrieving articles.

**(i) Article filtering results.** A total of 3,040 articles were identified from the initial search of the databases (PubMed, Web of Science, EMBASE, and the Cochrane Library), 2,002 articles were obtained after removing duplicates by EndNote 2020 software, and 86 articles were selected by reading and initial screening. A total of 24 papers were not available in their original data after the authors were contacted. Finally, according to the inclusion criteria, 30 records in English were included by full-text screening. The literature screening process can be seen in Fig. 5.

**(ii) Basic information of the included literature and evaluation of methodological quality.** A total of 318 participants were included in this study, which were all healthy volunteers. Of these participants, 123 were male, 155 were female, and 40 did not provide sex information. All articles were case-control studies and were written in English. Collection and preservation methods adopted by included studies contained RNAlater, 70% ethanol, 95% ethanol, immediate freezing/snap-frozen at −80°C or in liquid nitrogen, no additives or no solution, fecal occult blood test (FOBT), fecal immunochemical test (FIT), filter-paper, dimethyl sulfoxide-ethylenediaminetetraacetic acid solution (DETA), DETA-NaCl, ethylenediamine tetraacetic acid (EDTA), placed on ice, PSP (Invitek) buffer, DNA/RNA shield, OMNIgene.Gut, preservation buffer (PB), Eppendorf tubes, dry swab, NBgene-Gut, RNAssist, PerkinElmer/Chemagen SEB lysis buffer, fecal nucleic acid preservative, homemade RNA preservative, and HEMA reagents, among others. Sample storage temperatures include room temperature (RT), 30°C, 20°C, 4°C, −4°C, −20°C, and −80°C. Sample preservation time periods include 1 day, 2 days, 3 days, 4 days, 5 days, 7 days, 14 days, 15 days, 1 month, 5 weeks, 60 days, and so on. Most of articles utilized 16S rRNA gene sequencing (6–30), and four articles utilized shotgun metagenomic sequencing (31–34). Only 2 articles had data with consistent sample preservation methods and days of preservation, so meta-analysis was performed on these 2 articles and descriptive analysis was done on the rest of the literature. The basic characteristics of the included studies are shown in Table 1.

**TABLE 1:** The main characteristics of the included studies^*a*^

Study	Country (ethnicity^*b*^)	Study design	Sample size (*n*)	Age (yrs)	Male sex (%)	Stool collection methods	Storage duration	DNA extraction method	Sequencing region	Bioinformatic analysis program	Database used for taxonomic classification
Moossavi et al. (19)	USA	Case control	8	Range, 25–40	0.0	Immediate freezing, −80°C; 95% ethanol (RT); Card (RT)	48 h	QIAamp DNA stool minikit	V4	QIIME (v1.8)	Greengenes reference database
Angebault et al. (6)	France	Case control	3	33, 39, 47	33.3	No additive within 2 h freezing + 1 mo at −80°C; RNAlater dilution before freezing + 1 mo at −80°C	1 mo	MoBio PowerLyzer PowerSoil1 DNA isolation kit; Protocol Q, International Human Microbiota Standards	V3-V4	R	Greengenes 13.5, Silva 128 and RDP classifier
Zouiouich et al. (29)	France	Case control	19	Means ± SD, 40.2 ± 8.43	21.1	(i) Immediate freezing at −80°C (all methods); (ii) 4°C refrigerator 7 days (OC-auto sampling tubes); (iii) 30°C 7 days (OC-auto sampling tubes); (iv) RT 7 days (Hemotrust tubes, one-step FOB tubes, specimen collection container A tubes); (v) RT 70.2 days (GenSaver specimen collection cards, GenCollect specimen collection cards)	7 d, 70.2 d	PowerFecal DNA isolation kit	V3-V4	R	SILVA v138 database
Byrd et al. (34)	USA (non-Hispanic white [*n* = 13, 86.7%])	Case control	15	Means ± SD, 37.5 ± 10.7 (Range, 22–53)	46.7	FOBT cards; FIT tubes; 95% ethanol; RNAlater	4 days	PowerMag soil DNA isolation kit	shotgun metagenomic sequencing		
Fouhy et al. (15)	Ireland	Case control	7	N/A	28.6	Snap frozen on dry ice (4 min) + frozen at −80°C; frozen at −80°C; fresh, <4 ah	7 days	QIAmp fast DNA stool minikit	V3-V4	QIIME	SILVA SSURef database release 111
Chen et al. (11)	China	Case control	5	Range, 23–28	100.0	Standard; Norgen; OMNIgene.Gut; RNAlater; CURNA; HEMA; DNA/RNA Shield	7 days	Bead-beating QIAamp PowerFecal DNA kit	V1-V2, V3-V4, V4	QIIME2 (2018.8)	SILVA 132 99% 16S rRNA gene reference database
Neuberger-Castillo et al. (21)	Luxembourg	Case control	2	N/A	N/A	Snap frozen at −80°C within 2 h postcollection; RT collection without stabilizer; P-085 ‘‘red tube’’; OMNIgene.Gut tubes; RNAlater; RNAssist; AquaStool; PerkinElmer/Chemagen SEB lysis buffer	24 h, 1 wk, 2 wk	Chemagic DNA blood 4k kit special H24	V3-V4	R	RDP reference database v11.5
Szopinska et al. (26)	The Netherlands	Case control	14	>18	50.0	Fresh frozen, 4°C; OMNIgene.Gut	0, 24 h, 7 days	QIAamp DNA minikit, PowerFecal DNA isolation kit	V3-V4	QIIME	Greengenes v13.8
Choo et al. (12)	Australia	Case control	1	N/A	N/A	−80°C Freezer; 4°C fridge; OMNIgene.Gut; RNAlater; TE buffer; no solution, RT	72 h	MoBio Powerlyzer Powersoil DNA isolation kit	V4	QIIME, v1.8.0	Greengenes database v13.8
Holzhausen et al. (16)	Taiwan	Case control	12	Means ± SD, 35.4 ± 3.1 (Range, 22–55)	33.3	No additives, 4°C; fresh frozen, −80°C	0 h, 6 h, 24 h, 48 h, 72 h, 96 h	Zymo gel DNA recovery kit	V4	mothur	GreenGenes vgg_13_8_99 database
Vogtmann et al. (35)	USA (non-Hispanic white [*n* = 47] and other [*n* = 5])	Case control	52	Means ± SD, 35.8 ± 10.2	34.6	95% ethanol; RNAlater; FIT; FOBT; no additive	0, 4 days	N/A	N/A	QIIME	Greengenes database v13.8
Park et al. (23)	Korea (Korean)	Case control	3	Range, 20–40	66.7	NBgene-Gut; OMNIgene.Gut; ethanol, 70%; RNAlater; no solution	2, 15, 30, 45, 65 days	QIAamp DNA stool MiniKit	V3-V4	QIIME 2	SILVA database v132
Lim et al. (18)	Korea (Korean)	Case control	3	mid-20s	N/A	Room temp with and without OMNIgene.Gut solution up to 3 wk	0, 7, 14, or 21 days	QIAamp DNA stool minikit	V3-V4	QIIME 2	Greengenes 13.8 database
Sinha et al. (25)	USA	Case control	20	Range, 23–54	37.5	No additive; RNAlater; 70% ethanol; EDTA; dry swab; pre/postdevelopment FOBT	0, 24 h, 96 h	PowerSoil DNA isolation kit	V3-V5	QIIME pipeline v1.7	Greengenes database v13.5
Dominianni et al. (13)	USA	Case control	3	N/A	33.3	FOBT card (RT); Eppendorf tubes (RT); RNAlater (RT); immediately frozen at −80°C (fresh frozen)	3 days	PowerLyzer PowerSoil DNA isolation kit	V3-V4	QIIME	RDP classifier method and the IMG/GG Greengenes database of microbial genomes
Lauber et al. (17)	USA	Case control	2	N/A	100.0	20°, 4°, −20°, and −80°C	3, 14 days	MoBio PowerSoil DNA extraction kit	V1-V2	N/A	RDPII taxonomy
Penington et al. (24)	Australia	Case control	6	Range, 35–70	50.0	OMNIgene.Gut devices, RT; screw cap tubes, −80°C; screw cap tubes, −20°C; screw cap tubes, +4°C; sterile jar, +4°C	6–24 h	MoBio PowerSoil kit	V4	QIIME v1.8.1	MOTHUR v1.38.1, Greengenes 13_08 database
Bundgaard-Nielsen et al. (8)	Denmark	Case control	3	N/A	N/A	DNA/RNA Shield; PSP buffer; RNAlater; −20°C domestic freezer; RT, −20°C, or 4°C	24 h or 72 h	QIAamp fast DNA stool minikit	V4	QIIME	MiDAS database v1.20
Wu et al. (33)	China	Case control	12	N/A	N/A	RT, 4°C, 20°, and −80°C; liquid nitrogen; PB	4 h, 1 day, 3 day, 1 wk, 2 wk, 4 wk	DNeasy PowerSoil kit	Shotgun metagenomic sequencing	N/A	N/A
Panek et al. (22)	Croatia	Case control	4	N/A	N/A	OMNIgene.Gut; RT	0 days, 14 days	Power fecal DNA isolation kit (Mo Bio), fast DNA SPIN kit for feces (MP Biomedicals), QIAamp fast DNA stool minikit (QIAGEN)	V3–V4	QIIME	GreenGenes database v13_8, May 2013
von Huth et al. (27)	Denmark	Case control	22	Range, 23–64	59.1	Filter-paper; fresh-frozen, −80°C	5 wk, 5 mo	QIAamp DNA stool minikit	V1–V2	R	N/A
Bartolomaeus et al. (7)	Germany (Caucasian)	Case control	27	Means ± SD, 37.5 ± 10.2 (Range, 18–60)	48.1	Immediate freezing with dry ice (naive sample); Zymo DNA/RNA Shield with dry ice; OMNIgene.Gut, RT; 95% EtOH, RT; RNALater, RT; Zymo DNA/RNA Shield, RT	5–7 days	QIAamp PowerFecal DNA kit	V3–V4	metadeconfoundR	GreenGenes, SILVA, HITdb
Franzosa et al. (32)	USA	Case control	8	N/A	100.0	Fresh frozen (control); RNALater, RT; 95% EtOH, RT	0, 48 h	Qiagen AllPrep DNA spin columns	Shotgun metagenomic sequencing	MetaPhlAn	N/A
Wu et al. (28)	USA (Black/African-American [*n* = 3]; white [*n* = 7])	Case control	10	Median age, 26.5 (Range, 20–61)	60.0	Fresh; immediate freezing at −80°C; ice for 24 h; ice for 48 h; PSP (Invitek) buffer, RT 48 h	0, 24 h, 48 h	QIAamp DNA stool Minikit, PSP spin stool DNA plus kit, and the MoBio Powersoil DNA isolation kit; cells were lysed by bead beating in the presence of hot phenol and then processed with the QIAamp DNA stool Minikit	V1–V2; 454/Roche pyrosequence analysis, V1–3, V3–5, and V6–9	QIIME	RDP
Ezzy et al. (14)	Australia	Case control	3	Means ± SD, 33.7 ± 7.6	100.0	−80°C, −20°, 4°C, RT, DETA, DETA-NaCl, ethanol	120 h	ISOLATE Faecal DNA Extraction kit, FavorPrep Stool DNA Isolation minikit, DNEasy Powerlyzer PowerSoil DNA isolation kit, QIAamp DNA Stool minikit, Human Microbiome Project, European MetaHIT	V3-V4	QIIME	Greengenes database v13.8
Cardona et al. (9)	Spain	Case control	4	N/A	N/A	Frozen; unfrozen; RT	3 h, 24 h, 48 h, 72 h, 14 days, 1 mo	N/A	V4	QIIME	Silva 108 release database
Voigt et al. (31)	Germany	Case control	7	Means ± SD, 34 ± 6	71.4	Fresh frozen; RNAlater (at RT or at +4 to 10°C); day 7, 5 subjects’ stool, +4~10°C, RT 1 wk; day 392, 3 subjects’ stool, +4°C, 24 h	0, 2, 7, 60, 392, 600 and 773 days	G’NOMEs kit	Library generation and whole-genome shotgun sequencing	MOCAT	A database consisting of 10 universal single-copy marker genes extracted from 3,496 NCBI reference genomes and 263 human gut metagenomes
Nagata et al. (20)	Japan	Case control	11	N/A	N/A	Fresh; immediate frozen; 4°C; 25°C	1, 3, 7 days	Enzymatic lysis method	V3-V4	R	RDP v10.27, CORE and a reference genome sequence database obtained from the NCBI FTP site
Chen et al. (10)	Taiwan	Case control	4	Mean, 33.5 (range, 31–40)	N/A	OMNIgene.Gut kit; Stratec stool collection tube with DNA stabilizer; immediate frozen at −80°C	72 h, 7 days	QIAmp fast DNA stool minikit	V3-V4	QIIME v1.9.1	Greengenes database v13.8
Hill et al. (30)	Ireland	Case control	20	Mean, 70 (range, 34–83)	45.0	Fresh sample; Geotek OMNIgene.Gut kit; RNAlater; 4°C, −80°C, RT	1 wk, 2 wk	QIAamp DNA Stool minikit + RBB extraction protocol	V4-V5	QIIME	RDP reference database

a N/A, not applicable; EtOH, ethyl alcohol; FIT, fecal immunochemical test; FOBT, fecal occult blood test; immediately frozen = fresh-frozen; PB, preservation buffer; DETA, dimethyl sulfoxide-ethylenediaminetetraacetic acid solution; EDTA, ethylenediamine tetra acetic acid; QIIME, Quantitative Insights into Microbial Ecology; MOCAT, metagenomic analysis toolkit; RDP, Ribosomal Database Project; NCBI, National Center for Biotechnology Information; RBB, repeated bead beating.

b Details on ethnicity were provided if reported.

Newcastle-Ottawa scale was used to assess the quality of all studies, and the results showed that the scores of 2 literature were 7, 16 were 8, and 11 were 9, indicating that all included studies were high-quality literature (Table 2).

**TABLE 2:** Newcastle-Ottawa scale for assessing the quality of the 29 included studies

Reference	Country	Selection (possible range, 0–4)	Comparability (possible range, 0–2)	Exposure (possible range, 0–3)	Total score (possible range, 0–9)
Moossavi et al. (19)	USA	4	2	3	9
Angebault et al. (6)	France	3	2	3	8
Zouiouich et al. (29)	France	4	2	3	9
Byrd et al. (34)	USA	4	2	3	9
Fouhy et al. (15)	Ireland	4	2	3	9
Chen et al. (11)	China	3	2	3	8
Neuberger-Castillo et al. (21)	Luxembourg	3	1	3	7
Szopinska et al. (26)	The Netherlands	3	2	3	8
Choo et al. (12)	Australia	3	2	3	8
Holzhausen et al. (16)	Taiwan	3	2	2	7
Vogtmann et al. (35)	USA	4	2	2	8
Park et al. (23)	Korea	3	2	3	8
Sinha et al. (25)	USA	4	2	3	9
Dominianni et al. (13)	USA	3	2	3	8
Chen et al. (10)	Taiwan	4	2	3	9
Lim et al. (18)	Korea	3	2	3	8
Lauber et al. (17)	USA	3	2	3	8
Penington et al. (24)	Australia	3	2	3	8
Bundgaard-Nielsen et al. (8)	Denmark	3	2	3	8
Wu et al. (33)	China	3	2	3	8
Panek et al. (22)	Croatia	3	2	3	8
von Huth et al. (27)	Denmark	4	2	3	9
Bartolomaeus et al. (7)	Germany	4	2	3	9
Franzosa et al. (32)	USA	3	2	3	8
Wu et al. (28)	USA	4	2	3	9
Ezzy et al. (14)	Australia	4	2	3	9
Cardona et al. (9)	Spain	3	2	3	8
Voigt et al. (31)	Germany	4	2	3	9
Nagata et al. (20)	Japan	3	2	3	8
Hill et al. (30)	Ireland	4	2	3	9

**(iii) Storage time of ≤2 days.** A summary of the main characteristics of the included studies is presented in Table S1 in the supplemental material. Preservation at room temperature for 3 h did not affect the observed taxa compared with samples immediately frozen at −80°C (“gold standard”). No statistical differences were observed for *Bacteroides*, *Prevotellaceae*, and *Bifidobacterium* (9). Storage temperature had little effect on the microbial community during 4 h of temporary storage (33). Eight literatures studied the differences between different preservation methods and the gold standard for 24-h storage (8, 9, 16, 21, 24–26, 28). Among them, one reported that the 70% ethanol group had low microbial stability (25), and eight studies showed that there is no statistical difference in alpha diversity in 24-h and 48-h preservation groups of different preservation methods compared with the gold standard (8, 9, 16, 19, 21, 23, 24, 26). Six studies showed that beta diversity had no difference (8, 19, 21, 23, 25, 28), whereas one study reported that beta diversity decreased when preserved in OMNIgene.Gut for 6 to 24 h (24), and beta diversity changed the most during the first 24 h at 4°C (16). Furthermore, the impacts of preservation conditions are smaller than the difference between individuals. Seven studies showed that the bacterial composition was relatively stable after preservation for 3 h, 4 h, 24 h, and 48 h (9, 16, 21, 25, 28, 31–33). In brief, the composition of the microbial community was relatively stable when the feces were stored at room temperature for up to 24 h (9). The most prevalent phyla detected in all preservation conditions were *Firmicutes* and *Bacteroidetes*. Three studies found changes in several phylum- and genus-level bacterial taxa when samples were stored for 24 h. Compared with immediately frozen (−80°C) samples, samples stored in OMNIgene.Gut tubes (RT) showed an increase in *Lentisphaerae*, *Bacteroidetes*, and *Cyanobacteria* (24, 26) and a decrease in *Actinobacteria* (26), and the relative abundance of *Faecalibacterium* increased and that of *Alistipes* decreased in the preservation buffer (8). Under the condition of preservation for 48 h, the abundance of *Actinobacteria* of samples stored in the specimen collection card (RT) increased (19); the abundance of *Bacteroidetes* was significantly different from that in the gold standard in 70% ethanol (RT) group, while the *Veillonellaceae* and *Enterobacteriaceae* abundances were significantly different from those in the gold standard in the OMNIgene.Gut tubes (RT) group (23).

**(iv) Storage duration of ≥3 days and ≤7 days.**The alpha diversity of most of the samples preserved for 3 days, 4 days, or 5 days was similar to that of immediately frozen (−80°C) samples (8–10, 13–18, 21, 25, 28, 31, 35). Similarly, principal-coordinate analysis (PCoA) or nonmetric multidimensional scaling (NMDS) analysis showed that clustering of samples from the same individual is more evident than preservation conditions or preservation duration (7–18, 20, 25, 26, 29, 31, 33–35), suggesting that individual differences are more remarkable than differences caused by different preservation methods. The study found that the stability of 95% ethanol preservation is poor and the intraclass correlation coefficient (ICC) is lower and more diverse (34). Compared with samples stored at −80°C, storage at room temperature without additives for 3 days, storage in RNAlater for 3 days, and storage in DNA/RNA shield for 7 days resulted in a decrease in Shannon diversity index and evenness (11, 12, 31); samples preserved in PB (RT) had decreased Shannon and Simpson indexes (31). FOBT card caused an increase in observed OTUs and Shannon diversity index, whereas 95% ethanol did the opposite (20). One study found that within clusters of the same individual’s samples, the samples are distributed along the *y* axis according to the preservation methods (12). Bartolomaeus et al. (7) found that compared with dry ice, the alpha diversity of samples preserved in 95% ethanol and OMNIgene.Gut was significantly lower, and all preservation methods, especially RNAlater, 95% ethanol, and OMNIgene.Gut in beta diversity, were significantly different from dry ice.

The bacterial composition of most samples after storage for 3 to 5 days was not significantly different from that of samples immediately frozen (−80°C) (7, 16, 21, 25, 31). The main bacteria at the phylum level are *Firmicutes*, *Actinobacteria*, and *Bacteroidetes*. Some studies have shown that adding stabilizers can change the microbial community of samples. In samples preserved in PB, the relative abundance of *Faecalibacterium* increased and the relative abundance of *Alistipes* decreased (8); *Anaerostipes* significantly changed in the 4°C freezer, RNAlater, Tris-EDTA (TE) buffer, and no-additives (RT) group; and the relative abundance of *Bacteroides* increased and that of *Bifidobacteria* was reduced in the RNAlater (3 days, 7 days) and TE buffer (3 days) group (11, 12). However, *Bifidobacteria* abundance increased in no-additives (RT) preservation (12). *Sutterella* and *Faecalibacterium* abundances were significantly changed in OMNIgene.Gut stored for 3 days (12); the abundances of *Faecalibacterium* and *Cyanobacteria* were elevated and those of *Bifidobacterium* and *Bacteroides* decreased at 7 days (18, 26). FOBT cards, RNAlater, and 95% ethanol storage increased *Actinobacteria* and decreased *Verrucomicrobia* abundances compared with FIT tubes without adding solution (20). Two studies found the lowest ICC for the relative abundance of *Actinobacteria*, *Bacteroidetes*, and *Firmicutes* in samples preserved with 95% ethanol and FOBT cards, and the overall distribution differences in the relative abundance of each species in samples preserved without a solution are more significant than those caused by other methods (e.g., FIT and RNAlater) (34, 35). When samples were stored for 5 days, Ezzy et al. (14) found that the relative abundance of Pseudomonas and *Solibacillus* increased in samples stored at room temperature compared with that at −80°C. However, stabilizer-added samples resulted in an increased relative abundance of *Bifidobacteria*, *Faecalibacteria*, *Fusobacteria*, *Prevotella*, and *Roseburia* (11). When samples stored at room temperature, 30°C, and −80°C were compared, the relative abundance of *Blautia* decreased significantly at room temperature and *UGC-002*, *Faecalibacterium*, *Roseburia*, and *Ruminococcus* were unstable at 30°C (29).

**FIG 6:**
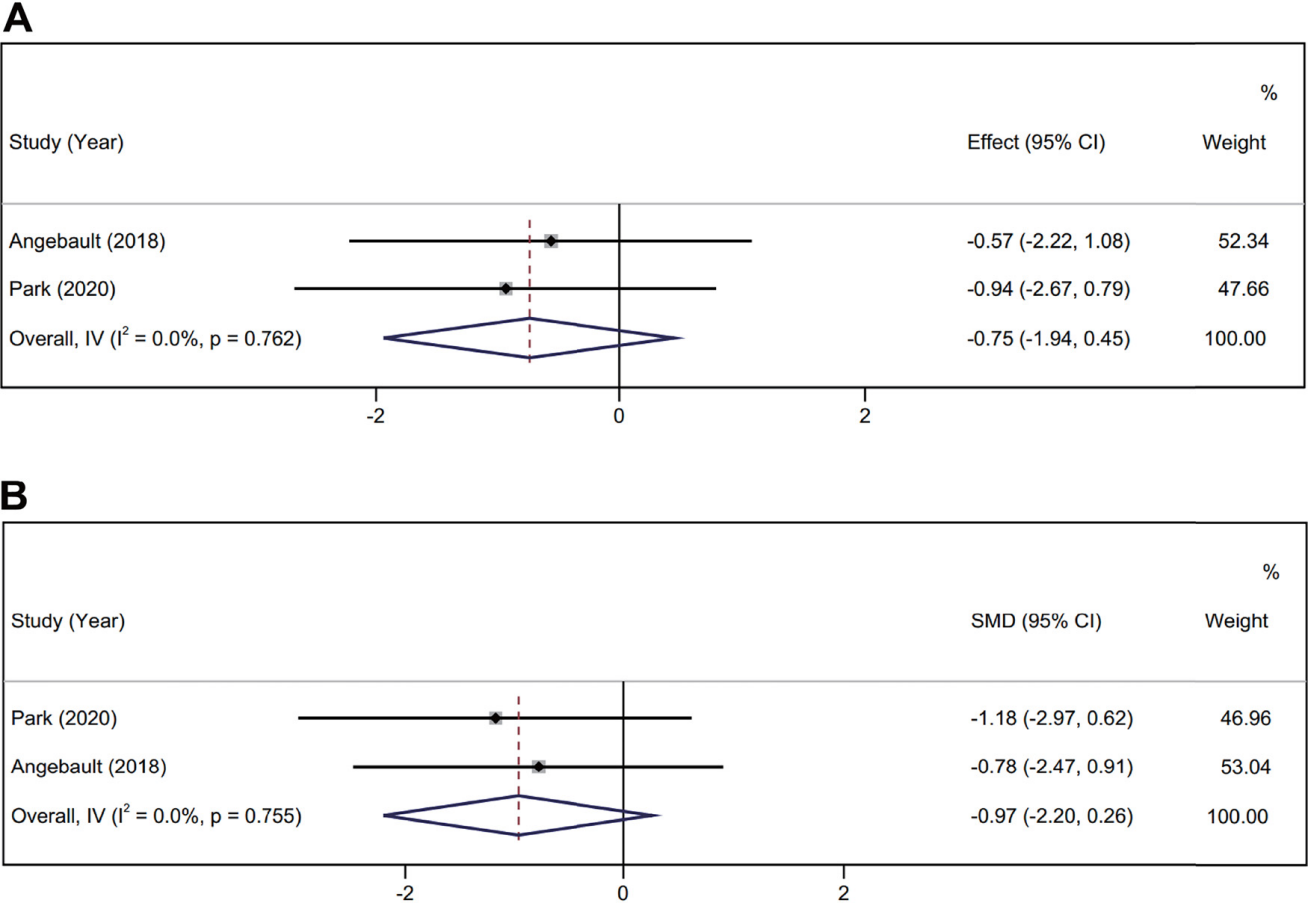
Alpha diversity of samples preserved for 1 month in RNAlater. (A) Shannon diversity index. (B) Simpson index.

**(v) Storage duration of >7 days.** Statistically, we analyzed data from two articles (including Shannon and Simpson indices) (6, 23). The heterogeneity test results showed no significant statistical heterogeneity between the two studies (I^2^ = 0.0%, *P* = 0.762; I^2^ = 0.0%, *P* = 0.755). The fixed-effect model was used to combine the data. The Shannon diversity index of samples stored in RNAlater for 1 month was not statistically significant compared with immediate freezing (standardized mean difference [SMD] = −0.75; 95% confidence interval CI, −1.94, 0.45; *P* = 0.220), and the Simpson index was also not statistically significant (SMD = −0.97; 95% CI, −2.20, 0.26; *P *= 0.123) (Fig. 6).

One study found that room temperature conditions significantly affected bacterial proportions only after being preserved for 2 weeks (9). Only a 0.36% difference was statistically significant between days 7 and 392 between RNAlater preservation and immediately frozen samples. All sample clustering and alpha diversity for the seven subjects showed little temporal variability and were less than the difference between fecal samples donors (31). Several studies have shown that there is no significant difference in alpha diversity between fecal specimens stored for 14 days, 15 days, and 21 days compared with that of samples immediately cryopreserved (9, 17, 18, 21, 30); beta diversity was closely associated with sample source (17, 18, 21, 22). A study using a preservation buffer for 14 days found a decrease in alpha diversity (33); the distribution distance between samples stored at room temperature and storage in liquid nitrogen is farther than that between preservation buffer and storage in liquid nitrogen, and the distance ratio is about 2:1, indicating that the difference in microbial composition between samples stored at room temperature for 14 days and those stored in liquid nitrogen is more significant (33). When the preservation duration was >14 days, the index of alpha diversity and the Bray-Curtis distance of beta diversity increased with time, but there was no significant difference (23), and another study also found Bray-Curtis distances rising over time in PCoA plots (18). Furthermore, one study examined the difference between filter paper preservation and immediate freezing, and they found a significant difference in alpha diversity results for samples stored on filter paper for 5 weeks versus 5 months and samples with a Bray-Curtis difference greater than 5 weeks after 5 months of storage (27).

The main bacteria at the phylum level are still *Firmicutes* and *Bacteroidetes*. Compared with frozen samples, the relative abundances of *Bifidobacterium* and *Faecalibacterium* increased in samples stored at room temperature without preservation solution for 2 weeks (9), and samples stored in OMNIgene.Gut at room temperature also showed increased *Bifidobacterium* and decreased *Bacteroides* abundances (18). The ratio of *Bacteroidetes*/*Lachnospiraceae* was raised, and the relative abundance of *Prevotella* was reduced on day 14 compared with that of day 0 in samples without preservation solution at −20°C (22). Moreover, one study by unsupervised clustering analysis of samples revealed that for each sample donor, genera with a high relative abundance were not affected by preservation solutions at different periods (e.g., 15 days, 30 days, 45 days, and 65 days) (23). In this study, the results of the fecal microbiota structure of the different donors were partially inconsistent. Still, the *Bacteroidetes* abundance in 70% ethanol was significantly different from that of the control group, and the overall relative abundance of the samples preserved in 70% ethanol and RNAlater were significantly different from that of controls (23). In a study from days 0 to 21, the relative abundance of partial bacteria at the genus level increased or decreased over time, and the change in the composition of samples in OMNIgene.Gut preservation solution was less than that in samples without preservative solution added at room temperature (18). The abundance of *Proteobacteria* in the samples stored directly at −80°C decreased in the 5th month. It was found that *Anaerostipes* and *Blautia* abundances were significantly different at 5 weeks, and the abundance of *Fusicatenibacter* had significant differences when stored at 5 months when comparing all the preservation methods (27).

## DISCUSSION

In the study of microbial communities, 16S rRNA genes are widely used in the phylogenetic, taxonomic, and diversity research of microorganisms. The phylogenetic results based on 16S rRNA information are very similar to those based on genome-wide information (36). To evaluate the effect of each preservation method, 16S rRNA sequencing of stool samples can be used to indirectly measure the quality of the samples and the characteristics of the microbial community (37). Therefore, the preservation method and storage quality of stool samples are crucial for researching gastrointestinal microbiota.

Currently, fecal DNA preservation is achieved by removing or inhibiting nucleases by adding buffer or lowering the temperature. Preservation methods adopted for microbial studies include freezing conditions (38), or they can be assisted by preservatives, such as ethanol (39), RNAlater (40), and mixtures of glycerol and phosphate-buffered saline (PBS) (41). Different preserving methods of samples can lead to significant changes in bacterial abundance. First, temperature is an essential factor in preserving fecal samples. Freezing leads to an increase in the *Firmicutes* to *Bacteroidetes* ratio. In contrast, room temperature preservation generally yields the opposite result, probably because Gram-positive bacteria maintain higher DNA stability than Gram-negative bacteria under freezing conditions (42). Low-temperature cryopreservation has always been the gold standard for sample preservation. Rapid freezing (−80°C, liquid nitrogen, or dry ice) is more beneficial for sample preservation as the temperature drops quickly, preserving the integrity of the cells by reducing ice crystal formation. However, 4°C is favorable for fungal growth and, therefore, is not recommended for preserving stool samples. In addition, preservatives/buffer/stabilizers can preserve fecal microbiota characteristics, and preserving samples without stabilizers can lead to an increase in specific taxa (e.g., OTUs of *Enterobacteriaceae*). Ethanol can inactivate nucleases by penetration and is a commonly used preservative. Different concentrations (70% and 95%) of ethanol, however, have different preservation effects. Notably, 70% ethanol leads to an increase in Streptococcus spp. and Haemophilus spp. Fecal samples are dehydrated in anhydrous ethanol to prevent DNA degradation; room temperature is the preferred temperature for sample preservation in anhydrous ethanol, which has been shown to keep DNA from human and canine fecal specimens for up to 8 weeks at room temperature (43). The deficit of ethanol is inflammability, which is not suitable for mailing specimens. A buffer containing EDTA (TE buffer) can inhibit the proliferation of certain microorganisms. However, experiments by Choo et al. (12) demonstrated that TE preservation is unstable. RNAlater could better tolerate freeze-thaw cycles but does not stabilize microbiota characteristics well after long periods of storage at room temperature (44). The meta-analysis showed that the alpha diversity of the samples stored in RNAlater for 1 month had no significant change compared with the samples stored at −80°C, suggesting that RNAlater has an acceptable effect on preserving the alpha diversity of samples within 1 month. However, a larger sample size is needed for future analysis. PB buffer, OMNIgene.Gut, and FTA card/FOBT card are more appropriate for room temperature storage. The glycerol-PBS mixture showed good preservation of bacterial intracellular DNA at −80°C. Hence, glycerol can be used as a protective agent for cryopreservation.

To more thoroughly investigate the best method of stool sample preservation, the following five different preservation methods were designed in this study: room temperature + anhydrous ethanol, liquid nitrogen + anhydrous ethanol, liquid nitrogen, −80°C refrigerator + anhydrous ethanol, and −80°C refrigerator. The addition of glycerol to frozen samples reduces cell damage and helps cells remain viable after freezing, so we added 10% glycerol as a protective agent to cryopreservation groups before cooling. In this experiment, we found the possibility of DNA degradation in long-term storage at room temperature despite dehydration by adding anhydrous ethanol. The PCoA analysis indicated that the results of the bacterial flora of the cryopreserved samples were relatively close, and the storage at room temperature led to the dispersion of the bacterial microbiota. Meanwhile, through the analysis of different taxa between groups, it can be seen that only the room temperature group and the fresh samples have different bacterial genera at the four time points detected, and the difference is statistically significant. There were differences in the bacterial genus among the samples in the third and sixth months in the −80°C refrigerator group, but they were not statistically significant.

Additionally, the alpha and beta diversity analysis showed that the species diversity, richness, and bacterial structure of the room temperature samples differed from those in the fresh samples and other experimental groups. The cryopreservation group was clustered with fresh samples in terms of relative abundance and diversity of flora. Nevertheless, whether adding absolute ethanol as a storage agent could make the microbial flora more stable and whether adding absolute ethanol and directly freezing samples will lead to differences in microbial communities have not been fully demonstrated in the four cryopreservation groups of our experiment. By comparing the five preservation methods in this experiment, we found that the optimal stool preservation protocol is −80°C/liquid nitrogen plus 10% glycerol, which can preserve stool samples with high quality for up to 12 months.

In conclusion, here, we presented a 12-month study of fecal sample preservation, and our study provides an empirical reference about experimental details for long-term high-quality storage of fecal samples in the field of gut microbiology research. The results showed that the combination of −80°C/liquid nitrogen deep cryopreservation and 10% glycerol was the most effective method for the preservation of stool samples and was suitable for long-term storage for at least 12 months. Both of these characteristics made the microbiota of the samples suitable for a wide range of subsequent experimental studies and analyses. The addition of anhydrous ethanol to the deep cryopreserved samples did not make a significant difference in the preservation of fecal microbiological characteristics. Our systematic review and meta-analysis showed that the preservation methods preservation solution and FOBT cards were relatively stable in terms of the structure, composition, and diversity of the preserved samples in short-term sample storage and were suitable for short-term preservation at room temperature.

## MATERIALS AND METHODS

### Ethics declarations.

Informed consent to participate in research was obtained.

### New original data.

**(i) Sample collection and preservation.** Fecal samples were collected from three healthy volunteers. Fresh fecal samples from the same time were transferred to the specimen collection box and immediately divided into sterile vials, with each tube of 200 mg divided into the following five groups for preservation: (i) −80°C refrigerator group, (ii) liquid nitrogen tank group, (iii) −80°C refrigerator + absolute ethanol group, (iv) liquid nitrogen + absolute ethanol group, and (v) room temperature + absolute ethanol group. We added 10% glycerol to the cryopreservation group as a protective agent before cooling.

**(ii) Devices and reagents.** Fecal DNA extraction kits were purchased from Beijing Kangwei Century Biotechnology Co., Ltd.; MiSeq kits were purchased from Illumina (USA). Anhydrous ethanol and glycerol were purchased from Shanghai Sinopharm Chemical Reagent Co., Ltd.

**(iii) DNA detection methods.** Before preservation, 1 tube was randomly selected from each group of samples for immediate testing, which were documented as month 0 (fresh samples). The samples were stored in groups and taken out for testing the corresponding indexes in the 3rd, 6th, 9th, and 12th months.

DNA was extracted using a DNA extraction kit, and bacterial 16S rRNA sequencing was performed with the Illumina MiSeq sequencing platform. The V3-V4 hypervariable region of the 16S rRNA gene was amplified from genomic DNA using the upstream forward primer 341F (5′-CCTACGGGNGGCWGCAG-3′) and downstream primer 805R (5′-GACTACHVGGGTATCTAATCC-3′). Each initial PCR system was prepared as required, consisting of DNA amplification premix, primer 341F (0.1 μM), primer 805R (0.1 μM), and DNA template (12.5 ng). Reactions were run in a T100 PCR thermocycle (BIO-RAD) according to the following cycling program: 3 min of denaturation at 94°C, followed by 18 cycles of 30 s at 94°C (denaturing), 30 s at 55°C (annealing), and 30 s at 72°C (elongation), with a final extension at 72°C for 5 min. A second PCR was carried out as follows: mix diluted amplicon (2 μL) with reaction solution containing DNA amplification premix, 0.5 μM fusion forward primer, 0.5 μM fusion reverse primer, and 30 ng of target DNA (total volume, 50 μL) and perform PCR with the same cycling procedure as above (cycle number 12).

**(iv) Data processing.** Fastq-files were demultiplexed by the MiSeq controller software (Illumina Inc.). The sequences were trimmed for amplification primers, diversity spacers, and sequencing adapters; merge paired; and quality filtered by USEARCH (45). UPARSE was used for operational taxonomic unit (OTU) clustering equaling or above 97% (46). The taxonomy of the OTUs was assigned, and sequences were aligned with the Ribosomal Database Project (RDP) classifier (47). The OTUs were analyzed by phylogenetic and OTU methods in the Quantitative Insights into Microbial Ecology (QIIME) software version 1.9.0 (48). Alpha diversity (observed OTU number, Shannon index, and Simpson index) and beta diversity (unweighted UniFrac distances and weighted UniFrac distances) measures were calculated based on the rarefied OTU counts. The variability of the flora among the four experimental groups at different periods with fresh samples was compared by differences in alpha diversity indexes across the groups. The species richness index indicated the abundance of each species; the Shannon diversity and Simpson index reflected microbial diversity, while the species rank clustering curve reflected species abundance and evenness. Principal-coordinate analysis (PCoA) through dimensionality reduction was constructed based on weighted and unweighted UniFrac distance matrices to identify potential principal components impacting changes in sample community composition. Meanwhile, samples were clustered based on UniFrac distance.

**(v) Statistical analysis.** All experimental data were analyzed by SPSS 20 statistical software, and the Wilcoxon signed-rank test was used to find the differential taxa between groups. A *P* value of <0.05 was considered statistically significant. GraphPad Prism 8 software was used to plot the obtained data. Adobe Illustrator 2021 software was utilized for graphic representation.

### Systematic review and meta-analysis with previous studies.

Our study has been registered in PROSPERO (registration identifier [ID] CRD42022328028), and the current study was prepared based on the meta-analysis of observational studies in epidemiology (MOOSE) guidelines (49).

**(i) Search strategy for studies.** The electronic databases PubMed, Web of Science, EMBASE, and the Cochrane Library were retrieved using a combination of both medical subject headings (MeSH) terms and free-text terms searches to collect published observational studies on the effect of stool sample preservation methods on intestinal microbiota, with reference lists of the included literature being traced. The retrieval period was from inception to April 7, 2022, and the language was limited to English. The search terms were as follows: (storage OR collection OR preservation OR store OR Cryopreservation OR Cryofixation OR Cryonic Suspension OR Cryonic Suspensions OR Suspension, Cryonic OR Suspensions, Cryonic) AND (microbiota OR microflora OR flora OR bacteria OR bacterial OR microbiome OR microorganism OR feces OR stool OR fecal) AND (gut OR intestinal OR bowel OR gastrointestinal) AND (diversity OR abundance OR richness).

**(ii) Criteria for inclusion and exclusion.** Inclusion criteria were as follows: (i) the study subjects were healthy volunteers aged ≥18 years, (ii) different stool specimen preservation methods were used to treat the specimens, (iii) the article outcome indicators were intestinal flora diversity and comparative relative abundance of intestinal flora, and (iv) the study type was an observational study.

Exclusion criteria were as follows: (i) study subjects with any acute or chronic diseases and under drug treatments or study subjects with animals or infants; (ii) inaccessible raw data or incomplete data; (iii) duplicate publication of data with poor quality; (iv) inaccessible full text; (v) reviews, conference abstracts, errata, among others; and (vi) studies unrelated to the purpose of this study.

**(iii) Article selection and data extraction.** This study used EndNote 2020 software to manage the literature. After checking the weight to remove duplicates, reading the titles and abstracts to remove articles that did not meet the inclusion criteria, and then reading the full text to remove articles that did not meet the inclusion criteria, we extracted the data from the final included articles.

Data extraction consisted of two parts. General information of the original literature was extracted, as follows: authors, year of publication, country, type of study, number of study subjects, age, sex, interventions (e.g., fecal preservation time, fecal preservation method), and method of determination of results. The outcome measures of interest were extracted, as follows: intestinal microbiota diversity and the relative abundance of intestinal microbiota. Two researchers performed all processes and cross-checked independently, and a third party decided whether to include the literature for controversial cases. If the literature lacked data, we contacted the authors by email to obtain the original information.

**(iv) Quality of article assessment.** This study employed the Newcastle-Ottawa scale (NOS) (50) for the quality assessment of the articles. The score was divided into the following three main sections: (i) selection of study subjects, with a total of 4 points; (ii) comparability between groups, with a total of 2 points; and (iii) assessment of outcomes and exposures, with a total of 3 points. The total rating was 9 points, with ≤3 points considered low-quality literature, 4 to 6 points considered moderate quality literature, and ≥7 points as high-quality literature. Two researchers completed all processes and cross-checked independently, and a third party decided whether to include controversial literature.

**(v) Data analysis and synthesis.** Stata (version 14.0 MP; StataCorp, College Station, USA) was used to analyze the extracted data. Continuous variables were expressed as standardized mean differences (SMDs) and 95% confidence intervals (95% CI). If the heterogeneity was small (*P ≥ *0.1; I^2^, ≤50%), a fixed-effects model was used for the pooled analysis, and if the heterogeneity between studies was considerable (*P* < 0.1; I^2^, >50%), a random-effects model was selected for the joint analysis. Sensitivity analysis was performed by changing the data analysis model to test the stability of the meta-analysis results. If the literature number was greater than 10, publication bias analysis was used. Qualitative descriptions of the literature were performed for those with insufficient data for meta-analysis. A difference was judged to be statistically significant if a *P* value was <0.05.

### Data availability.

All data generated or analyzed during this study are included in this published article.
