# Supplementary material for: Effects of Stool Sample Preservation Methods on Gut Microbiota Biodiversity: New Original Data and Systematic Review with Meta-Analysis
Source: Microbiol Spectr. 2023 Apr 24;11(3):e04297-22. doi: 10.1128/spectrum.04297-22 (PMC10269478; doi:10.1128/spectrum.04297-22)
Supplement: Supplemental file 2 — Supplemental material. Download spectrum.04297-22-s0002.pdf, PDF file, 0.05 MB [file spectrum.04297-22-s0002.pdf]

Search strategy

#1 AND #2 AND #3 AND #4
#4 ((diversity[Title/Abstract]) OR (abundance[Title/Abstract])) OR (richness[Title/Abstract])
#3 (((gut[Title/Abstract]) OR (intestinal[Title/Abstract])) OR (bowel[Title/Abstract])) OR (gastrointestinal[Title/Abstract])
#2 (((((((((microbiota[Title/Abstract]) OR (microflora[Title/Abstract])) OR (flora[Title/Abstract])) OR (bacteria[Title/Abstract])) OR (bacterial[Title/Abstract])) OR (microbiome[Title/Abstract])) OR (microorganism[Title/Abstract])) OR (feces[Title/Abstract])) OR (stool[Title/Abstract])) OR (fecal[Title/Abstract])
#1 ((((storage[Title/Abstract]) OR collection[Title/Abstract]) OR preservation[Title/Abstract])) OR (((((((store[Title/Abstract]) OR Cryopreservation[Title/Abstract]) OR Cryofixation[Title/Abstract]) OR Cryonic Suspension[Title/Abstract]) OR Cryonic Suspensions[Title/Abstract]) OR Suspension, Cryonic[Title/Abstract]) OR Suspensions, Cryonic[Title/Abstract])
